# MRI segmentation of head and neck tumors using hybrid attention mechanism and dense dilated spatial pyramid pooling

**DOI:** 10.1002/acm2.70426

**Published:** 2025-12-29

**Authors:** Qiang Han, Songlin He, Yuebin Zheng, Huacai Zhong, Dengyao Luo, Jun Wu, Zhiqiang Zhao, Bincheng Yan, Chengjian Cao, Xiu Liu

**Affiliations:** ^1^ Intelligent Perception and Control Key Laboratory of Sichuan Province Sichuan University of Science and Engineering Yibin China; ^2^ School of Automation and Information Engineering Sichuan University of Science and Engineering Yibin China; ^3^ Department of Otorhinolaryngology Head and Neck Surgery Zigong First People's Hospital Zigong China

**Keywords:** deep learning, densely connected atrous spatial pyramid pooling, head and neck tumor segmentation, MRI, spatial–channel dual attention, U‐Net

## Abstract

**Background:**

Head and neck cancer (HNC) involves anatomically intricate regions where precise target delineation is essential for radiotherapy. The superior soft‐tissue contrast of MRI provides clearer boundary visualization compared with computed tomography (CT), enabling tighter margins and supporting daily plan adaptation in online adaptive radiotherapy. However, despite the advances of U‐Net‐based deep learning models, tumor segmentation in HNC remains challenging due to ill‐defined borders and heterogeneous intensity patterns, which limit feature extraction and compromise small‐lesion recognition.

**Purpose:**

To overcome the limitations of traditional approaches, this study proposes an improved SCDU‐Net model that integrates collaborative spatial‐channel attention mechanisms with densely connected atrous spatial pyramid pooling techniques, aiming to significantly enhance the segmentation accuracy and robustness of head and neck tumor MRI images.

**Methods:**

SCDU‐Net integrates two modified modules to improve segmentation capability. The model incorporates a spatial‐channel dual attention module (SC) in the decoding pathway, which strengthens critical tumor feature expression through adaptive channel weight adjustment mechanisms, while capturing long‐range spatial dependencies using coordinate axis attention to improve localization accuracy of small target lesions. Additionally, the network embeds a densely connected atrous spatial pyramid pooling module dense atrous spatial pyramid pooling (DenseASPP) in the bottleneck layer, which enhances edge contour detail perception through multi‐scale receptive field fusion strategies, improving the network's segmentation performance.

**Results:**

Our proposed model is evaluated on the publicly available HNTS‐MRG2024 dataset, showing promising results compared to existing approaches.

**Conclusions:**

The results indicate that by integrating two modules, our method performs spatial and channel feature recalibration and multi‐scale contextual modeling within the deep neural network, yielding more accurate and promising head and neck tumor segmentation with potential to assist physicians in diagnosis.

## 1 INTRODUCTION

Head and neck cancer (HNC) ranks as the fifth most common malignant tumor globally and the eighth leading cause of cancer death, primarily caused by factors such as smoking, alcohol abuse, and viral infections.^[^ ^1^ ^]^ This group of cancers exhibits diverse disease entities and complex pathological types, primarily encompassing three major categories: (1) Tumors arising from mucosal surfaces; (2) Tumors of the thyroid and salivary glands; (3) Relatively rare primary tumors originating in other head and neck organs.^[^ ^2^ ^]^ Furthermore, radiotherapy (RT) serves as a primary treatment modality for HNC, with over 40% of cancer patients worldwide receiving RT at least once; its therapeutic efficacy is closely associated with the accurate delineation of the gross tumor volume (GTV).^[^ ^3^ ^]^ Moreover, MRI (Magnetic Resonance Imaging) is a critical noninvasive imaging technique that is extensively utilized for anatomical sites such as the brain, nasopharynx, and oropharynx; owing to its superior soft‐tissue contrast, MRI has become an essential complement or alternative to computed tomography (CT).^[^ ^4^ ^]^ Accurate MRI‐based tumor segmentation enables clinicians to optimize treatment planning by providing precise anatomical guidance for radiation dose delivery and critical structure avoidance.^[^ ^5^ ^]^

Currently, the delineation of head and neck tumor contours typically relies on manual contouring by experienced radiologists. However, this process is associated with several limitations, including time‐consuming procedures, poor reproducibility, and significant inter‐ and intra‐observer variability.^[^ ^6^ ^]^ Therefore, developing an automated segmentation technique holds substantial academic and clinical value for quantitative analysis and assessment of head and neck tumors and normal tissues.^[^ ^7^ ^]^

With the rapid development of deep learning, convolutional neural networks have progressed from foundational models like CNN^[^ ^8^ ^]^ to advanced architectures such as VGGNet,^[^ ^9^ ^]^ FCN,^[^ ^10^ ^]^ U‐Net,^[^ ^11^ ^]^ ResNet,^[^ ^12^ ^]^ and DenseNet,^[^ ^13^ ^]^ achieving impressive results across various computer vision tasks. Leveraging the powerful feature representation abilities of deep networks, their applications in medical image analysis have expanded rapidly.^[^ ^14^ ^]^ In particular, deep learning has led to notable advances in automatic segmentation of head and neck tumors in MRI. Among these methods, U‐Net stands out for its symmetrical encoder–decoder design and skip connections, enabling the simultaneous capture of high‐level semantic context and fine‐grained local features. This architecture has shown excellent segmentation performance and is widely regarded as a benchmark in the field.^[^ ^15^ ^]^ However, during the down‐sampling process, successive convolution operations impair the accurate delineation of small tumors. In the up‐sampling phase, although skip connections help recover spatial information, cascaded convolutions and nonlinear activations may lead to high‐level feature degradation, thereby limiting the final feature map resolution. Residual networks address these issues by mitigating degradation, deepening the model, and improving the extraction of detailed features. Additionally, spatial–coordinate attention modules enhance spatial awareness by assigning weights to different regions and modeling long‐range dependencies along horizontal and vertical axes. These modules suppress irrelevant background signals and compensate for segmentation errors caused by large lesion variability. Dense atrous spatial pyramid pooling further improves multi‐scale feature fusion by capturing contextual information at multiple receptive fields. Integrating these three components into the U‐shaped architecture effectively addresses key challenges in both down‐sampling and up‐sampling stages, enhancing overall segmentation performance.

Therefore, this study proposes SCDU‐Net (Spatial‐Channel Collaborative Dense U‐Net), potentially leading to improved segmentation of primary tumors (GTVp) and metastatic lymph nodes (GTVn) in head and neck MRI. Our model is evaluated on the publicly available HNTS‐MRG2024 dataset, one of the few publicly accessible head and neck datasets providing MRI modality.^[^ ^16^ ^]^

The contributions of this work include: (1) Development of SCDU‐Net, an adapted U‐Net architecture that combines spatial‐channel attention mechanisms with Dense ASPP modules for head and neck tumor MRI segmentation; (2) Integration of spatial–channel attention in the down‐sampling pathway to enhance feature representation and improve small target detection; (3) Implementation of Dense ASPP at the bottleneck to capture multi‐scale contextual information for better boundary delineation; along with a hybrid loss function combining Dice and Focal loss is employed to address class imbalance challenges.

The remainder of this paper is organized as follows. Section 2 reviews related work and recent developments. Section 3 describes the proposed method in detail. Section 4 presents the experimental setup and analyzes the results. Section 5 discusses the limitations of the model and outlines future research directions.

## 2 RELATED WORKS

In recent years, researchers have systematically addressed the performance limitations of U‐Net in medical image segmentation through structural enhancements. These enhancements target three critical aspects: feature extraction optimization during down‐sampling, detail recovery improvement in up‐sampling, and information flow facilitation via dense connectivity.

During the down‐sampling stage, conventional U‐Net architectures often suffer from spatial information loss, which compromises lesion detection accuracy. To mitigate this limitation, Hu et al.^[^ ^17^ ^]^ integrated spatial attention mechanisms with multi‐scale down‐sampling modules to enhance model sensitivity to ambiguous boundary regions. Khan et al.^[^ ^18^ ^]^ introduced residual‐based down‐sampling units to strengthen low‐resolution feature representation. For capturing richer contextual information, Peng et al.^[^ ^19^ ^]^ incorporated graph attention mechanisms to model global dependencies within local features, thereby improving segmentation of complex tumor morphologies. Additionally, Wang et al.^[^ ^20^ ^]^ proposed adaptive dynamic convolution that adjusts receptive fields and down‐sampling weights, enabling fine‐grained image detail preservation.

During the up‐sampling stage, recovering small‐scale tumor structures and lymph nodes poses significant challenges. Li et al.^[^ ^21^ ^]^ designed boundary‐aware modules combined with hierarchical feature fusion to enhance spatial detail restoration. Wang et al.^[^ ^22^ ^]^ introduced progressive feature reconstruction pathways that refine feature decoding iteratively, improving small lesion detection capabilities. Rahman et al.^[^ ^23^ ^]^ employed cascaded attention‐guided decoders that suppress background noise while reinforcing target responses, enhancing boundary continuity, and completeness. Furthermore, Li et al.^[^ ^24^ ^]^ proposed multi‐scale residual attention mechanisms that effectively boost segmentation performance in low‐contrast regions.

For feature propagation enhancement, dense connection structures have gained prominence in medical segmentation networks. Chen et al.^[^ ^25^ ^]^ combined dense connectivity with residual learning to promote deep feature reuse and improve convergence stability. Song et al.^[^ ^26^ ^]^ developed hybrid sparse–dense block architectures that optimize the balance between segmentation performance and parameter efficiency. Lin et al.^[^ ^27^ ^]^ introduced multi‐branch dense fusion networks that capture multi‐scale features through parallel pathways, improving adaptability to structurally complex tumors. Moreover, Muksimova et al.^[^ ^28^ ^]^ integrated attention mechanisms with dense blocks, achieving robust feature representation. This approach is particularly effective for challenging head and neck tumor segmentation tasks characterized by fuzzy boundaries and heterogeneous morphologies.

## 3 METHODS

### 3.1 Residual network

In the imagenet large scale visual recognition challenge (ILSVRC), several landmark architectures have achieved breakthrough performance that fundamentally advanced deep learning for computer vision. Specifically, AlexNet (8 layers)^[^ ^29^ ^]^ in 2012, VGG‐19 (19 layers) in 2014, and GoogLeNet (22 layers)^[^ ^30^ ^]^ in 2014 progressively demonstrated that strategic increases in network depth, when properly implemented, substantially improve feature extraction capabilities and classification accuracy. These architectures established the foundation for understanding the relationship between network depth and representational power in convolutional neural networks.

However, indefinitely increasing network depth does not yield proportional performance improvements. Excessively deep architectures become susceptible to gradient‐related issues, including vanishing and exploding gradients, which destabilize the training process and ultimately degrade segmentation accuracy. This limitation becomes particularly pronounced in medical imaging applications, such as head and neck tumor MRI segmentation, where the inherent complexity of anatomical structures, significant inter‐ and intra‐lesion variability, and subtle tissue contrast variations impose additional constraints on feature learning, thereby amplifying the adverse effects of training instabilities in deep networks.

**FIGURE 1:**
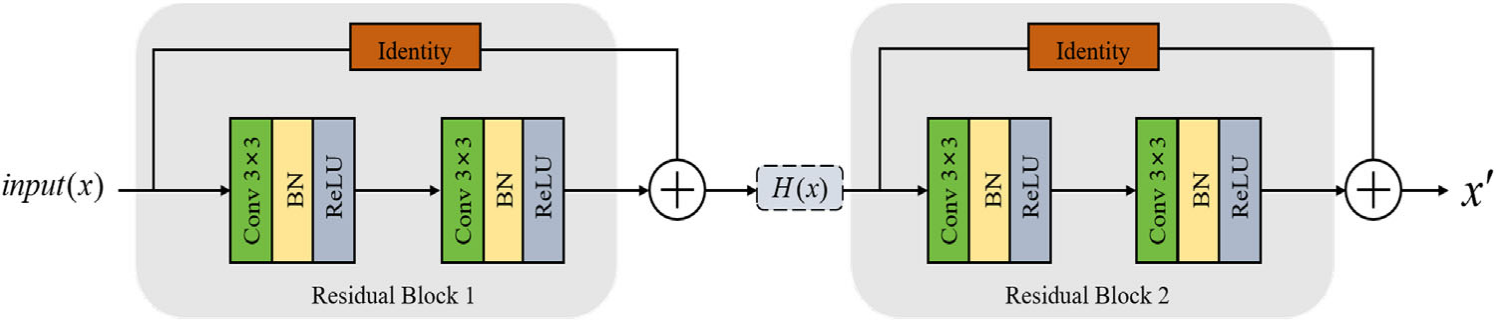
Diagrams of the cascaded residual network architecture of the encoder module. It comprises two consecutive residual blocks, each containing two layers of 3 ×3 convolution operations, batch normalization, and ReLU activation, with independent identity connections.

To address this issue, residual connections are introduced into the encoder module, replacing the conventional stacked convolutional layers in the original network, as illustrated in Figure 1. When networks approach accuracy saturation, incorporating cascaded residual blocks with identity shortcut connections enables further network deepening without performance degradation. Consider an input denoted as x. The proposed architecture consists of two consecutive residual blocks connected in series. In the first residual block, the input features x undergo processing through two consecutive 3 ×3 convolutional layers, each followed by batch normalization (BN) and ReLU (Rectified Linear Unit) activation, yielding a residual function $F_{1}\left(x\right)$. An identity mapping is integrated via a skip connection that directly passes x to the addition operation, producing the intermediate result $H\left(x\right)=x+F_{1}\left(x\right)$. The intermediate feature H(x) subsequently serves as input to the second residual block, where it passes through another pair of 3×3 convolutional layers, each followed by BN and ReLU activation, to obtain the second residual function $F_{2}\left(H\left(x\right)\right)$. A second identity mapping directly passes H(x) to the final addition operation, producing the final feature representation $x^{\prime }$. This computational process is formalized in Equation (1):

(1) $$x^{\prime }=H\left(x\right)+F_{2}\left(H\left(x\right)\right)=x+F_{1}\left(x\right)+F_{2}\left(x+F_{1}\left(x\right)\right).$$

Rather than learning direct mappings, each residual block learns the residual functions $F_{1}\left(x\right)$ and $F_{2}\left(H\left(x\right)\right)$, respectively. During training, these residuals naturally converge toward zero, enabling increased network depth without accuracy degradation. As shown in Figure 1, this cascaded residual architecture effectively increases the network depth of the encoder module to 20 layers, further enhancing the model's feature extraction capability, while ensuring that critical information is preserved during each down‐sampling stage through dual identity connections.

### 3.2 Spatial and channel dual attention network

**FIGURE 2:**
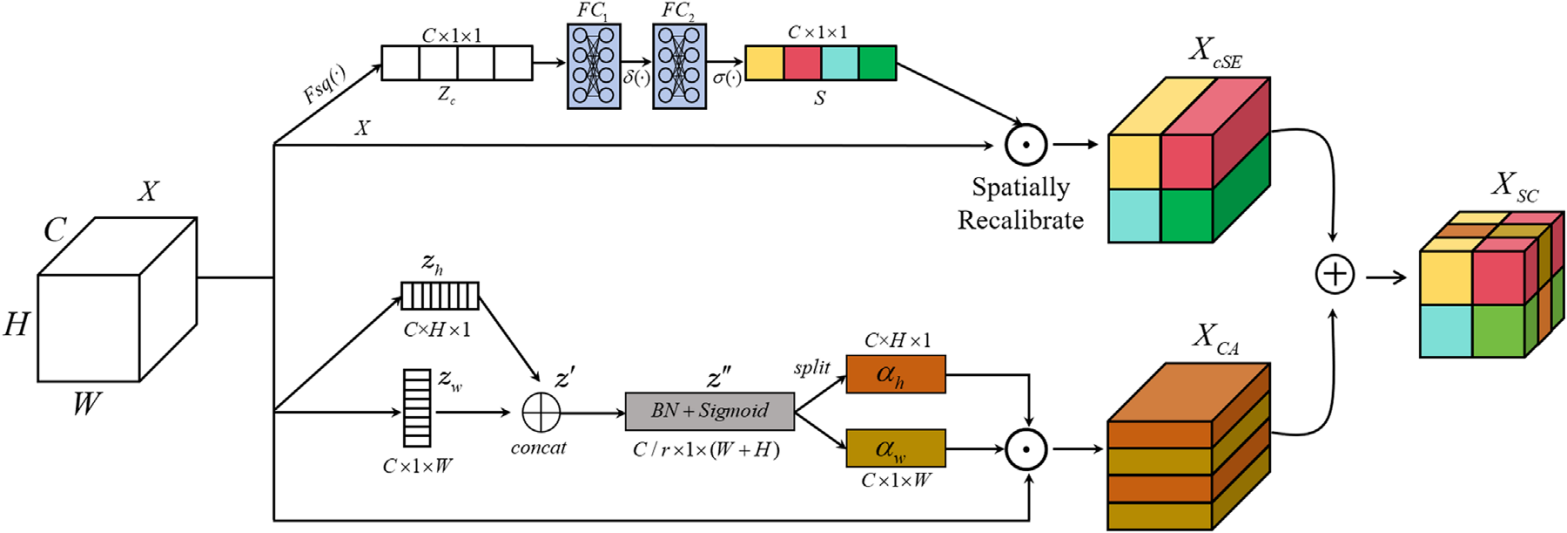
Schematic diagram of the spatial and channel attention (SC Attention) module. The upper branch extracts channel‐wise weights via global pooling and fully connected layers, while the lower branch employs coordinate attention to generate direction‐aware spatial maps. The two outputs are fused through element‐wise addition. SC, spatial and channel attention.

The segmentation of GTV presents significant challenges due to substantial variations in lesion shape and size, coupled with high intensity similarity between lesion regions and surrounding normal tissues.^[^ ^31^ ^]^ While residual neural networks demonstrate competence in extracting lesion‐specific features, they exhibit limited capability in capturing global contextual information within medical images, potentially leading to information loss or feature distortion in critical lesion regions. To address these limitations and enhance spatial information processing, we propose a spatial‐channel dual attention (SC) module, based on the Concurrent Spatial and Channel Squeeze & Excitation (csSE) architecture.^[^ ^32^ ^]^ The SC module is specifically designed to strengthen feature representation by effectively exploiting spatial positional information and modeling global dependencies, as illustrated in Figure 2.

#### 3.2.1 Channel attention branch

Attention mechanisms and their derivative architectures have emerged as fundamental components in modern computer vision systems, demonstrating substantial improvements in feature extraction and representation across diverse tasks including image classification, object detection, and image segmentation, while simultaneously driving significant progress in cross‐modal applications.^[^ ^33^ ^]^ To enhance the network's capacity for discriminative feature learning and address the aforementioned challenges in medical image segmentation, this study integrates the SENet architecture, originally proposed by Hu et al.,^[^ ^32^ ^]^ as a core component of our proposed framework.

Unlike the spatial attention mechanism (sSE), which primarily focuses on modeling spatial position information, the channel attention mechanism (cSE) emphasizes capturing interdependencies among features in the channel dimension to enhance the model's expressive capability. This module introduces global information compression and channel recalibration mechanisms, enabling the network to dynamically assign weights to each channel based on loss feedback during training. This process highlights key features while suppressing invalid or redundant information, effectively mitigating information loss caused by neglecting inter‐channel differences in convolution and pooling operations. We place this module after the residual network to better allocate weights following feature extraction.

The upper branch of Figure 2 illustrates the structure of cSE: assuming the feature after the convolution operation is $X\in R^{C\times H\times W}$ after the convolution operation, where C denotes the number of channels, H and W represent the height and width, respectively. Global average pooling $F_{sq}\left(\cdot \right)$ is applied to each channel independently to generate channel‐wise statistics. For the cth channel (c=1,2,…,C), the operation is defined in Equation (2):

(2) $$Z_{c}=F_{sq}\left(X_{c}\right)=\frac{1}{H\times W}\sum_{i=1}^{H}\sum_{j=1}^{W}X_{c}\left(i,j\right),$$

where $X_{c}\in R^{H\times W}$ represents the cth channel of the input feature map X. Next, the resulting channel‐wise statistics $Z={\left[Z_{1},Z_{2},\text{…},Z_{C}\right]}^{T}\in R^{C\times 1\times 1}$ are passed through two fully connected layers (the first layer denoted as $FC_{1}$ for dimensionality reduction, and the second as $FC_{2}$ for dimensionality expansion) to yield the channel‐wise attention weights $S\in R^{C\times 1\times 1}$. The computation is specified in Equation (3):

(3) $$S=\sigma (FC_{2}\left(\delta \left(FC_{1}\left(Z_{c}\right)\right)\right)),$$

where σ denotes the sigmoid activation function, and δ denotes the ReLU activation function. $FC_{1}$ and $FC_{2}$ represent the first fully connected layer for dimensionality reduction and the second fully connected layer for dimensionality expansion, respectively. Finally, the channel attention weights S are used to recalibrate the original feature map X, obtaining a reweighted feature map with properly assigned channel weights through element‐wise multiplication via broadcasting. The operation is given in Equation (4):

(4) $$X_{cSE}=S⊙X,$$

where ⊙ denotes element‐wise multiplication, and $X_{cSE}\in R^{C\times H\times W}$ represents the channel‐recalibrated feature map.

#### 3.2.2 Spatial attention branch

The squeeze‐and‐excitation (SE) attention mechanism has gained widespread adoption in deep learning due to its exceptional performance in channel‐wise attention modeling, demonstrating remarkable effectiveness across image classification, object detection, and medical image segmentation tasks.^[^ ^34^ ^]^ To address the computational limitations of global average pooling inherent in SE blocks, Wang et al.^[^ ^35^ ^]^ proposed ECA‐Net (Efficient Channel Attention Network), which employs lightweight 1D convolutions to enhance inter‐channel interactions while maintaining computational efficiency.

Despite the proven effectiveness of channel squeeze‐and‐excitation (cSE) in channel‐wise attention modeling, its capacity for capturing fine‐grained local spatial features remains limited when applied independently. To overcome this limitation and fully exploit the complementary nature of attention mechanisms, we propose an integrated approach that combines spatial attention with channel SE mechanisms. Specifically, we adopt the coordinate attention (CA) mechanism proposed by Hou et al.,^[^ ^36^ ^]^ which has demonstrated superior performance in small object segmentation tasks.^[^ ^37^ ^]^ The CA mechanism captures long‐range spatial dependencies by decomposing global average pooling into separate operations along with the height and width dimensions, followed by lightweight convolutions that generate spatially aware attention maps with enhanced positional sensitivity. The lower branch of Figure 2 illustrates the structure of CA: given a feature map after convolution, global average pooling is performed separately along the vertical and horizontal directions, as defined by the following Equation (5):

(5) $$z_{h}=\frac{1}{W}\sum_{i=1}^{W}X\left(h,i,c\right),z_{w}=\frac{1}{H}\sum_{w=1}^{H}X\left(j,w,c\right).$$

After obtaining the height‐wise feature vector $z_{h}\in R^{C\times 1\times W}$ and the width‐wise feature vector $z_{w}\in R^{C\times H\times 1}$, they are concatenated and fused to generate the combined feature representation $z^{\prime }=z_{h}+z_{w}$. This combined feature is then passed through a shared convolution layer (with output channel dimension C/r) followed by a ReLU activation to obtain an intermediate representation $z^{\prime \prime }\in R^{(H+W)\times 1\times C}$. Subsequently, $z^{\prime \prime }$ is split into two branches corresponding to horizontal and vertical directions, which are separately processed by convolutional layers (with output channel dimension C) and a sigmoid activation function to generate attention weights as Equation (6):

(6) $$\alpha_{h}=\sigma \left(Conv2d\left(z_{h}^{\prime \prime },W_{h}\right)\right),\alpha_{w}=\sigma \left(Conv2d\left(z_{w}^{\prime \prime },W_{w}\right)\right),$$

where $W_{h},W_{w}\in R^{1\times 1\times C/r\times C}$. The resulting attention weights $\alpha_{h}$ and $\alpha_{w}$ are broadcast along the width and height dimensions, respectively, to match the shape of the original feature map $X\in R^{H\times W\times C}$. The attention‐enhanced feature map incorporating coordinate information is then computed via element‐wise multiplication; the Equation (7) is as follows:

(7) $$X_{CA}=X⊙\left(\alpha_{h}\times \alpha_{w}\right).$$

Finally, the outputs of the SE and CA modules are fused to produce the final spatial–channel attention feature map. This step can be expressed as Equation (8):

(8)

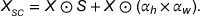




Notably, the CA mechanism is capable of capturing spatial dependencies along both horizontal and vertical directions, a capability that traditional spatial squeeze‐and‐excitation (sSE) lacks. Unlike sSE, which compresses spatial dimensions, CA preserves more precise positional information, thereby enhancing the model's ability to perceive structural details.

### 3.3 Densely connected atrous spatial pyramid pooling

Multi‐scale feature extraction constitutes a fundamental component for enhancing semantic segmentation performance, where receptive field design and computation critically determine the effectiveness of capturing contextual information across different scales.^[^ ^38^ ^]^ Atrous spatial pyramid pooling (ASPP) addresses this challenge by employing parallel atrous convolutions with varying dilation rates to expand receptive fields and capture multi‐scale contextual information.^[^ ^39^ ^]^ However, the conventional parallel architecture of ASPP exhibits inherent limitations in receptive field utilization efficiency and multi‐scale semantic feature correlation modeling.

**FIGURE 3:**
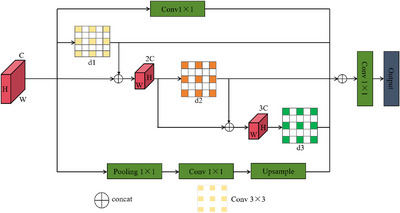
Diagram of the densely connected atrous spatial pyramid pooling network structure. It employs parallel atrous convolution branches with different dilation rates (d1=1,d2=2,d3=3) and applies pruning operations to the traditional network connections.

To overcome these constraints, we propose an enhanced dense atrous spatial pyramid pooling (Dense ASPP) architecture that optimizes the connectivity strategy among atrous convolutional layers. Unlike traditional ASPP, which concatenates outputs from all preceding layers, Dense ASPP employs a selective connection scheme where each layer receives inputs exclusively from: (1) the original input feature map, and (2) output features from layers with smaller dilation rates than the current layer. This strategic modification achieves dual benefits: enhanced receptive field expansion through hierarchical feature propagation and reduced computational overhead by eliminating redundant feature concatenations, as demonstrated in Figure 3.

In the improved Dense ASPP structure, the input to each layer no longer consists of a concatenation of feature maps from all preceding layers but includes only the original input feature and the output features from layers with dilation rates smaller than that of the current layer. For details, refer to Equation (9):

(9) $$x_{l}=H_{l}\left(\left[x_{0},\left\{x_{i}|i\in S_{l}\right\}\right]\right),$$

let $x_{l}$ denote the output feature of the lth layer, $H_{l}\left(\cdot \right)$ represent the nonlinear mapping function of the lth layer, and $x_{0}$ denote the original input feature map. The set $S_{i}=\left\{\begin{array}{c} i|i<l,d_{i}<l \end{array}\right\}$ represents the collection of layers, where the dilation rate is smaller than that of the current layer $d_{l}$, and $\left\{x_{i}|i\in S_{i}\right\}$ refers to the set of output features from these layers.

Table 1 illustrates the selective connection mechanism of Dense ASPP, where each layer l receives concatenated inputs from the original feature map and outputs of layers with smaller dilation rates, ensuring hierarchical multi‐scale feature integration. By restricting the feature connections, redundant or irrelevant information can be reduced. The size of the receptive field plays a crucial role in deep neural networks. In the case of spatial convolution, the dilation rate significantly affects the receptive field size. Generally, a larger receptive field enables the network to capture more context information. In this work, we design three convolutional layers with dilation rates $d_{1}=1,d_{2}=2$, and $d_{3}=3$, respectively. Given a kernel size K and dilation rate d, the theoretical receptive field RF of a dilated convolution can be expressed as Equation (10):

(10) RF=k+(k−1)(d−1).

**TABLE 1:** Pseudo‐code for Dense ASPP connectivity scheme.

Algorithm 1: Pseudo‐codes for Dense ASPP	
1:	Input original feature map $x_{0}$ and dilation rates $\left\{d_{1},d_{2},\text{…},d_{n}\right\}$;
2:	Initialize feature outputs $\{x_{1},x_{2},\text{…},x_{n}\}=\emptyset$;
3:	**for** l=1 to n:
4:	Determine set $S_{l}=\left\{i|d_{i}<d_{l}\right\}$;
5:	**if** $S_{l}=\emptyset$:
6:	$\text{input}\_\text{features}=x_{0}$;
7:	**else**:
8:	$\text{input}\_\text{features}=\text{Concatenate}(\left[x_{0},\left\{x_{i}|i\in S_{l}\right\}\right])$;
9:	**end if**
10:	$x_{l}=H_{l}\left(\text{input}\_\text{features}\right)$ with dilation rate $d_{l}$;
11:	**end for**
12:	Output Dense ASPP features $\left\{x_{1},x_{2},\text{…},x_{n}\right\}$;

Abbreviation: DenseASPP, dense atrous spatial pyramid pooling.

With our modifications to the connection structure, if two hollow convolutions are superimposed, the theoretical size of the maximum receptive field can be expressed as:

(11) $$K=K_{1}+K_{2}-1.$$

During the data preprocessing stage, the images were resampled to a fixed resolution of 448×448. After four down‐sampling operations in the encoder module, the spatial resolution was reduced to 32×32. According to Equations (10) and (11), the maximum theoretical receptive field is 13, which covers approximately 66% of the feature map area. This substantial coverage allows the network to effectively capture global contextual information while maintaining computational efficiency.

### 3.4 SCDU‐net

**FIGURE 4:**
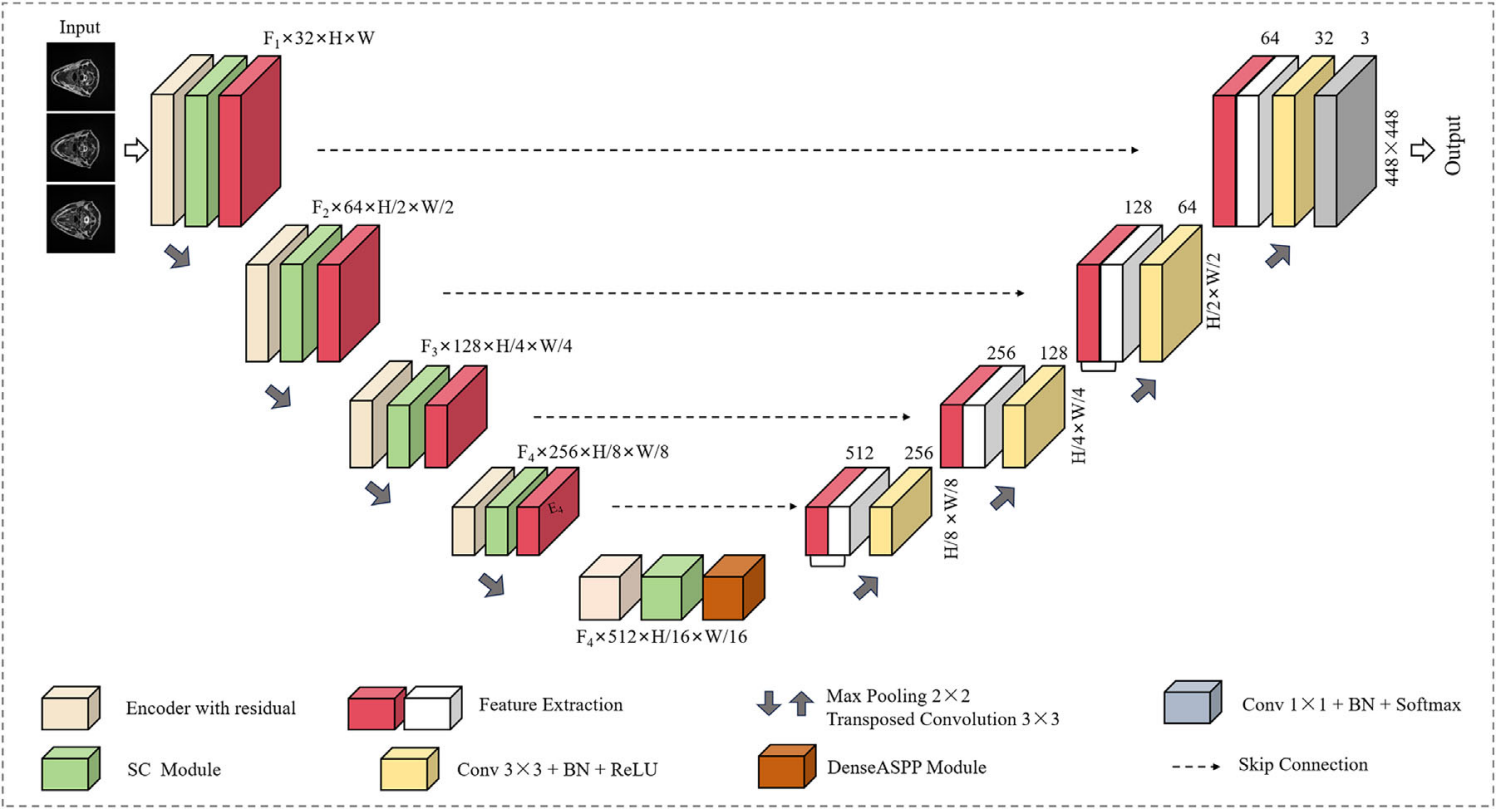
Structure diagram of SCDU‐Net. The model integrates residual blocks and spatial‐channel attention in the encoder and employs a Dense ASPP module in the bottleneck to enhance multi‐scale feature representation, enabling accurate segmentation of small tumors without increasing network depth.DenseASPP, Dense atrous spatial pyramid pooling.

In this study, we propose an improved deep learning model, termed SCDU‐Net, based on the classic U‐Net architecture, tailored specifically for head and neck tumor segmentation in MRI images. The model innovatively integrates key concepts such as residual networks, dilated convolutions, dense connectivity, and attention mechanisms. The overall network architecture is illustrated in Figure 4, where spatial dimensions and channel variations of each module are explicitly annotated.

The model adopts a typical encoder–decoder structure with a symmetric design, comprising four down‐sampling and four up‐sampling operations. In the encoding stage, spatial resolution is progressively reduced from 448×448 to 32×32 through hierarchical down‐sampling, while the number of feature channels is expanded to facilitate multi‐level feature extraction of tumor regions. In the decoding stage, the reverse process is applied, restoring spatial resolution while gradually reducing channel dimensions to reconstruct spatial details. To bridge the encoder and decoder, a Dense ASPP module is introduced in the bottleneck layer. By employing multiple atrous convolutions with different dilation rates, this module effectively captures multi‐scale contextual information of the lesion regions, enhancing robustness to variations in tumor shape and size. Moreover, to address class imbalance and boundary ambiguity, a composite loss function combining Dice loss and Focal loss is employed.

### 3.5 Combined loss function

To address the class imbalance problem in head and neck MRI tumor segmentation, we designed a combined loss function that integrates Focal Loss^[^ ^40^ ^]^ and Dice Loss. Focal Loss introduces a modulating factor $p_{t}=\exp\left(-CE\right)$ to standard cross‐entropy loss, dynamically adjusting the weights of samples with different difficulties. The calculation first obtains the cross‐entropy loss CE(p,y), then computes the predicted probability $p_{t}=\exp\left(-CE\right)$, as shown in Equation (12):

(12) $$L_{focal}={\left(1-p_{t}\right)}^{\gamma }\cdot CE\left(p,y\right),$$

where γ=2 is the focusing parameter controlling the down‐weighting rate of easy examples. Dice Loss is based on the Dice similarity coefficient, computing the overlap between predictions and ground truth labels in a differentiable manner. For multi‐class segmentation, the network output is first converted to probability distributions via Softmax, then the Dice coefficient for each class c is shown in Equation (13):

(13)

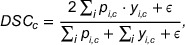

where $\varepsilon =10^{-8}$ is a smoothing term to avoid division by zero. The final Dice Loss is defined as shown in Equation (14):

(14)

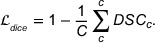

The combined loss function integrates both losses through linear weighting, as shown in Equation (15):

(15) $$L_{combined}=\alpha \cdot L_{focal}+\left(1-\alpha \right)\cdot L_{dice},$$

where α is a weighting parameter. We experimentally use α=0.7, meaning the ratio of Focal Loss to Dice Loss is 0.7:0.3. This combination leverages both advantages; the ability of Focal Loss to handle class imbalance effectively, while Dice Loss ensures better region consistency. Therefore, Focal Loss provides better classification boundary definition, while Dice Loss ensures better region consistency. To rapidly validate the effectiveness, we perform a grid search over the weight space with intervals of 0.1, evaluating each configuration across all cross‐validation folds. The tabulated results represent the averaged metrics over all validation folds. The results are shown in Table 2.

**TABLE 2:** Ablation study on weight allocation for combined loss function.

Focal weight (α)	Dice weight (1−α)	DSC ↑	HD95 ↓	Notes
1.0	0.0	0.8412	8.73	Focal only
0.8	0.2	0.8539	7.28	—
**0.7**	**0.3**	**0.8647**	**6.56**	**Optimal**
0.6	0.4	0.8591	6.94	—
0.5	0.5	0.8503	7.45	Equal weight
0.0	1.0	0.8376	8.92	Dice only

Abbreviations: DSC, Dice similarity coefficient; HD95, Hausdorff95.

*Note*: The bolded values represent the optimal weight ratios.

## 4 EXPERIMENT SETTINGS

### 4.1 Dataset and data preprocessing

#### 4.1.1 Dataset

**FIGURE 5:**
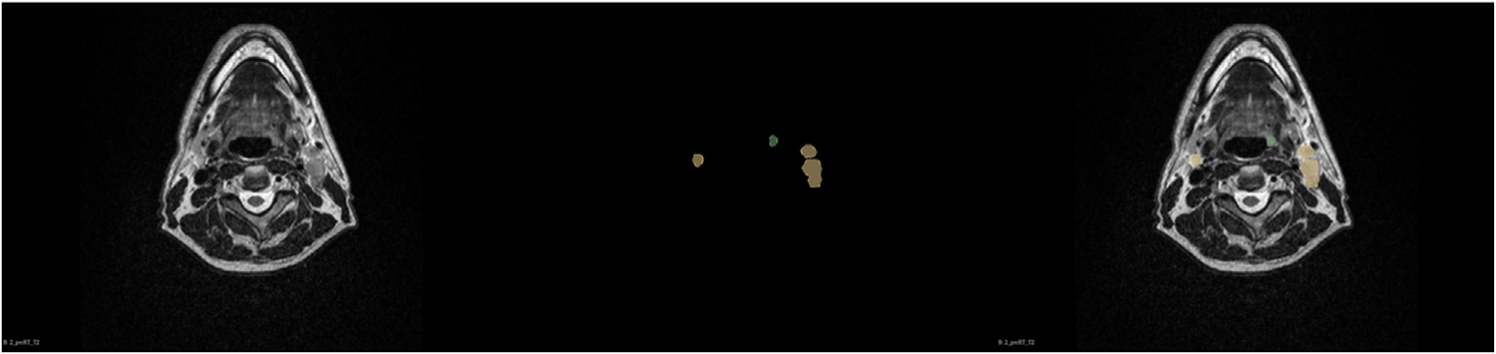
Example of the head and neck MRI data from a patient in the HNTS‐MRG. Green represents tumor GTVp, and yellow represents GTVn. GTVn metastatic lymph nodes; GTVp, primary gross tumor volume; HNTS‐MRG, head and neck tumor segmentation for MR‐guided applications.

This study utilized the head and neck tumor segmentation for MR‐guided applications (HNTS‐MRG) 2024 challenge dataset from the medical image computing and computer assisted intervention (MICCAI) challenge, specifically Task 1. The dataset comprises pre‐radiotherapy T2‐weighted MRI scans from 150 patients, intended for segmenting primary tumors (GTVp) and lymph node tumors (GTVn) in head and neck RT planning. All DICOM files were converted to NIFTI format, with most images maintaining a resolution of 512 ×512 pixels in the *XY* plane. The data were resampled while preserving this resolution. The segmentation masks designate primary tumors (GTVp) as value 1, lymph node tumors (GTVn) as value 2, and background as value 0, as shown in Figure 5. A detailed table describing the dataset is shown in Table 3.

**TABLE 3:** Detailed description of HNTS‐MRG 2024 public dataset.

Dataset characteristic	Description/Value
**Patient information**	
Total number of patients	150 (Publicly available data)
Patient population	Histologically proven head and neck cancer patients
Primary cancer types	Predominantly oropharyngeal cancer (OPC) and cancer of unknown primary
Treatment center	University of Texas MD Anderson Cancer Center (MDACC)
Treatment protocol	Radiotherapy (RT) with thermoplastic mask immobilization
**Imaging specifications**	
Scanner models	Mixed
Magnetic field strength	Not specified
Sequence type	T2‐weighted anatomical sequences
Fat suppression	Mixed (fat‐suppressed and non‐fat‐suppressed images)
Image format	NIfTI (.nii.gz), converted from anonymized DICOM
Anatomical coverage	Top of clavicles to bottom of nasal septum (oropharynx to shoulders)
Acquisition timepoints	Pre‐RT (1‐3 weeks before RT) and Mid‐RT (2‐4 weeks intra‐RT)
**Sequence parameters**	
Repetition time (ms)	4800 (1400‐6250)
Echo time (ms)	80 (74‐375)
In‐plane resolution (mm)	0.5 (0.4‐0.98)
Slice thickness (mm)	2.0 (1.0‐2.5)
Field of view	Cropped to consistent oropharynx region
**Segmentation information**	
Target structures	Primary gross tumor volume (GTVp) and metastatic lymph nodes (GTVn)
GTVp per patient	At most 1 (can be 0)
GTVn per patient	Variable number (can be 0)
Label values	Background = 0, GTVp = 1, GTVn = 2
Ground truth generation	STAPLE consensus from 3‐4 expert observers
Observer experience	Medical doctors with ≥2 years HNC segmentation experience
Faculty verification	Experienced radiation oncology faculty (>10 years experience)
**Tumor volume statistics (Based on the available dataset)**	
GTVp volume range	0.22–58.56 mL
GTVp median volume	8.22 mL
GTVp mean volume ± SD	12.15 ± 11.33 mL
GTVn volume range	0.38–126.77 mL
GTVn median volume	10.33 mL
GTVn mean volume ± SD	14.97 ± 17.18 mL
**Tumor location distribution**	
Primary sites	Predominantly oropharyngeal region
Nodal locations	Various neck levels (specific distribution not reported)
Anatomical subregions	Not specified

Abbreviations: GTVn metastatic lymph nodes; GTVp, primary gross tumor; volume; HNTS‐MRG, head and neck tumor segmentation for MR‐guided applications.

#### 4.1.2 Data preprocessing

**FIGURE 6:**
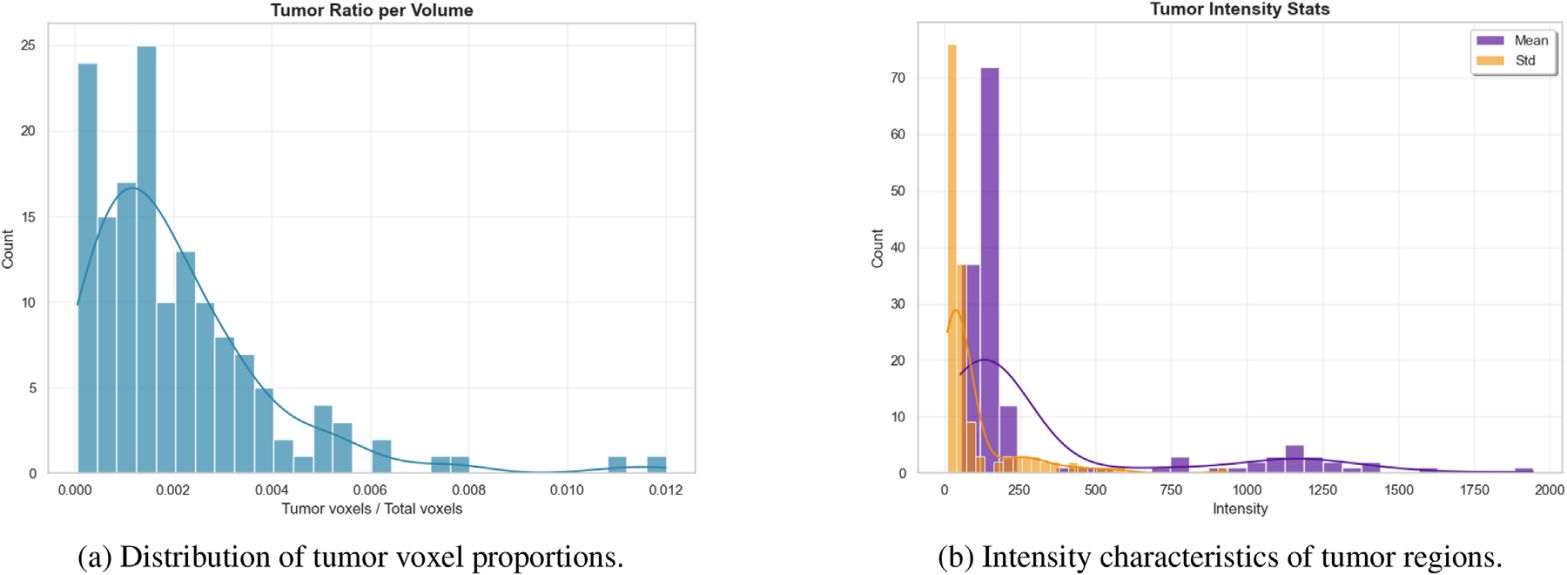
Quantitative analysis of the HNTS‐MRG 2024 dataset. Figure (a) illustrates the distribution of tumor voxel proportions, while Figure (b) depicts the intensity characteristics of tumor regions, with purple representing the mean and yellow representing the standard deviation. HNTS‐MRG, head and neck tumor segmentation for MR‐guided applications.

To comprehensively investigate the feature distribution of the dataset and develop appropriate preprocessing strategies, we conducted a systematic quantitative analysis of T2‐weighted MRI images and their corresponding tumor segmentation masks from all patients. First, we analyzed the distribution of tumor voxel proportions is shown in Figure 6a to evaluate the ratio of tumor to background regions. The results indicate that tumor voxel proportions in most cases range from 0.000 to 0.004, exhibiting a pronounced right‐skewed distribution, which highlights a significant class imbalance in the dataset. To address this issue, a composite loss strategy is used in Section 3.5 to mitigate its impact on segmentation performance.

Second, we performed a detailed analysis of intensity characteristics within tumor regions as shown in Figure 6b. The average intensity values of tumor regions are primarily distributed between 0 and 200, with a peak between 50 and 100, while the standard deviation of intensity is mainly concentrated between 0 and 50. These findings suggest that the signal intensity within tumor regions is relatively uniform, providing a foundation for subsequent intensity‐based preprocessing steps.

**FIGURE 7:**
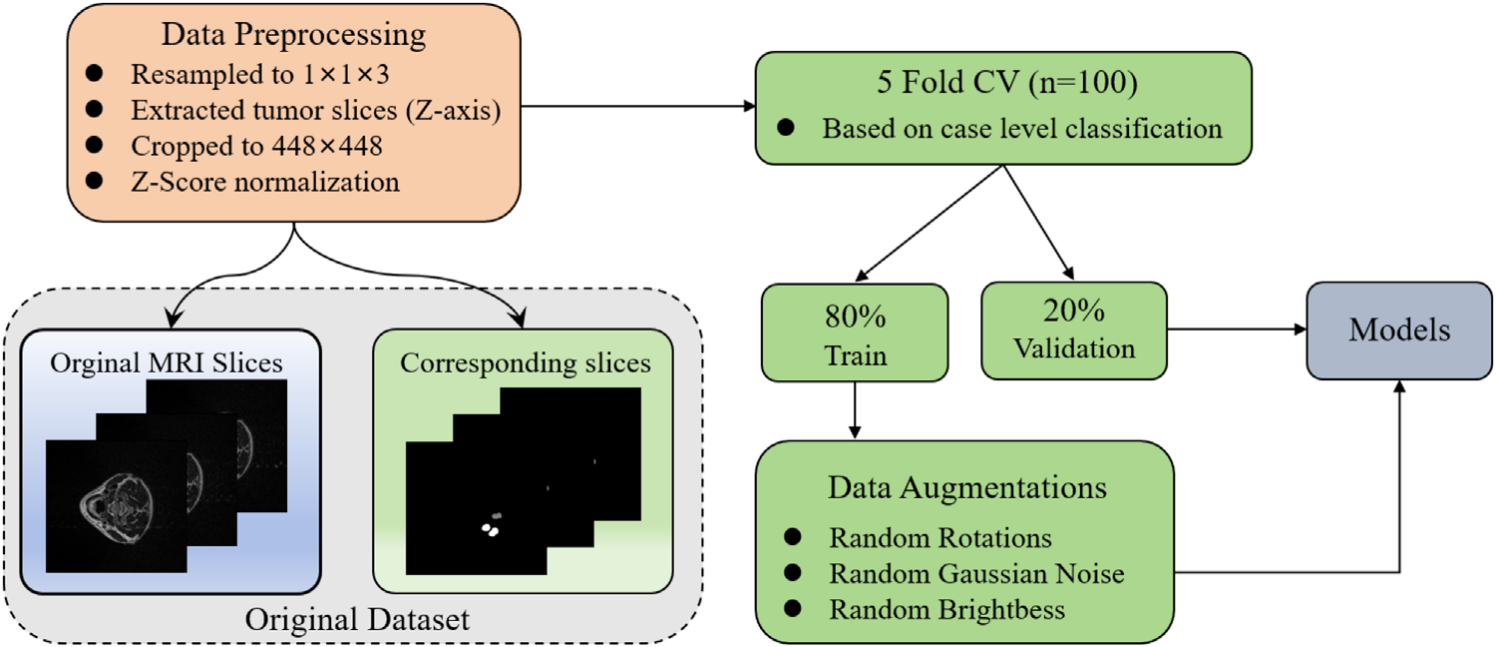
Flowchart of preprocessing and data partitioning for the HNTS‐MRG 2024 dataset. HNTS‐MRG, head and neck tumor segmentation for MR‐guided applications.

While deep learning algorithms exhibit excellent noise resistance capabilities, preprocessing procedures remain indispensable. The data processing pipeline encompasses several key stages: implementing intensity normalization through statistical analysis of valid pixel regions to achieve image standardization; uniformly resizing input images to 448×448 resolution; screening and retaining slices containing pathological regions. As the official online test set was not accessible, we performed five‐fold cross‐validation locally, with each fold splitting the data into training (*n* = 80) and validation (*n *= 20) sets. The final performance was reported as the average across all folds. In addition, an independent test set (*n *= 50) was partitioned for further evaluation.The preprocessing workflow is illustrated in Figure 7.

### 4.2 Evaluation metrics

In medical image segmentation, the Dice similarity coefficient (DSC) is widely used to quantify the overlap between predicted regions and ground truth labels. For a pair of binary arrays a and $\hat{a}$, the DSC is Equation (16) as:

(16) $$DSC\left(a,\hat{a}\right)=\frac{2\sum_{i}a_{i}{\hat{a}}_{i}}{\sum_{i}a_{i}+\sum_{i}{\hat{a}}_{i}},$$

here, $a_{i}$ and $\hat{a_{i}}$ denote the true and predicted labels for the ith voxel, respectively. When the target class is completely absent in the ground truth (i.e., all $a_{i}=0$), this metric becomes uninformative. To better capture performance across multiple samples, we employ the aggregated Dice coefficient ($DSC_{agg}$) as a global evaluation metric. Given a dataset $P={\left\{\left(a^{\left(n\right)},{\hat{a}}^{\left(n\right)}\right)\right\}}_{n=1}^{N_{P}}$ of ground truth and prediction pairs, the aggregated DSC is defined as:

(17) $$DSC_{\mathrm{agg}}\left(P\right)=\frac{2\sum_{n,i}a_{i}^{\left(n\right)},{\hat{a}}_{i}^{\left(n\right)}}{\sum_{n,i}a_{i}^{\left(n\right)}+\sum_{n,i}{\hat{a}}_{i}^{\left(n\right)}}.$$

This equation computes the Dice score after aggregating voxel‐level counts across all cases, providing a more reliable measure of segmentation performance, especially under class imbalance conditions. To comprehensively evaluate the boundary accuracy of RT target delineation, the 95% Hausdorff distance was employed as a supplementary evaluation metric. The Hausdorff distance quantifies the maximum deviation between two boundary contours, defined as:

(18) Hausdorff=max{h(P,Q),h(Q,P)},

where P denotes the pixel set of prediction pixels, and Q represents the pixel set of ground truth. For P and Q, which can be expressed as $\left\{p_{1},p_{2},\text{…},p_{n}\right\}$ and $\left\{q_{1},q_{2},\text{…},q_{n}\right\}$, both in Euclidean space, the $h\left(P,Q\right)=max_{p\in P}min_{q\in Q}||p-q||$.

### 4.3 Implementation details

All experiments were implemented in PyTorch on a system running Windows 11 with Python 3.10, CUDA 11.8, and cuDNN 9.1.0. The hardware platform consisted of a 12‐core 12th Gen Intel Core i7 processor, 32GB RAM, and an NVIDIA GeForce RTX 3090 GPU (24GB VRAM). Model optimization employed the Adam optimizer (initial learning rate: 0.0001, batch size: 16) for up to 1000 epochs, with a cosine annealing learning rate scheduler. An early stopping mechanism with 20‐epoch patience was incorporated to mitigate overfitting.

### 4.4 Ablation experiments

Experiments on the HNTS‐MRG dataset were conducted with residual connections in all models. As shown in Table 4, the ablation experiments demonstrate the individual and combined contributions of the proposed components. Adding the SC module alone to the baseline improved the average DSC by 8.5% and reduced the average Hausdorff95 distance by 2.54 mm. Incorporating the Dense module separately achieved an average DSC improvement of 9.15% and Hausdorff95 reduction of 2.81 mm. The complete proposed method, integrating both SC and Dense modules, achieved the best average performance with DSC of 0.8647 (+14.33%) and Hausdorff95 of 6.56 mm (‐34.1% reduction) compared to the baseline.

**TABLE 4:** Quantitative results of ablation experiments conducted on the self‐partitioned training dataset of HNTS‐MRG (all models use residual connections as baseline), with the best results in **bold**.

	DSC ↑						Hausdorff95 (mm) ↓					
Methods	Fold0	Fold1	Fold2	Fold3	Fold4	Average	Fold0	Fold1	Fold2	Fold3	Fold4	Average
Baseline	0.7456	0.7689	0.7523	0.7612	0.7534	0.7563	10.87	12.34	11.45	11.76	11.23	11.53
Baseline+SC	0.8123	0.8289	0.8176	0.8245	0.8198	0.8206	8.76	9.23	8.95	9.12	8.87	8.99
Baseline+Dense	0.8201	0.8298	0.8234	0.8287	0.8256	0.8255	8.54	8.89	8.67	8.78	8.71	8.72
Ours	**0.8523**	**0.8745**	**0.8612**	**0.8698**	**0.8657**	**0.8647**	**7.23**	**5.98**	**6.71**	**6.12**	**6.76**	**6.56**

Abbreviation: HNTS‐MRG, head and neck tumor segmentation for MR‐guided applications.

**FIGURE 8:**
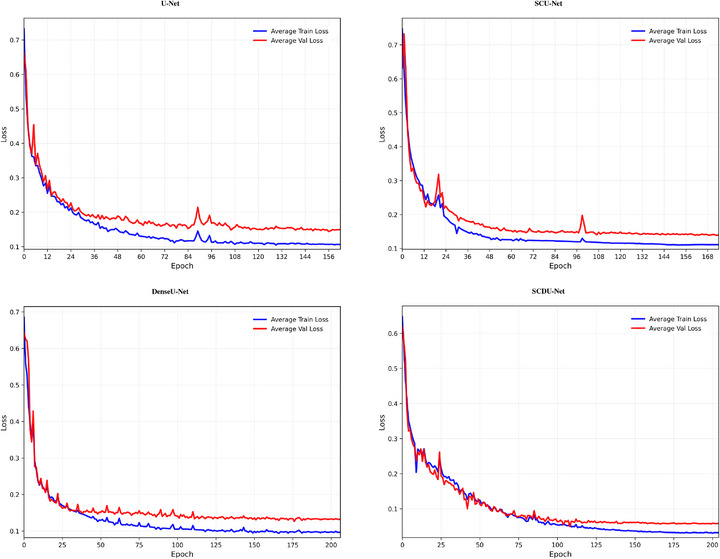
Loss curves for different network architectures during training.

**FIGURE 9:**
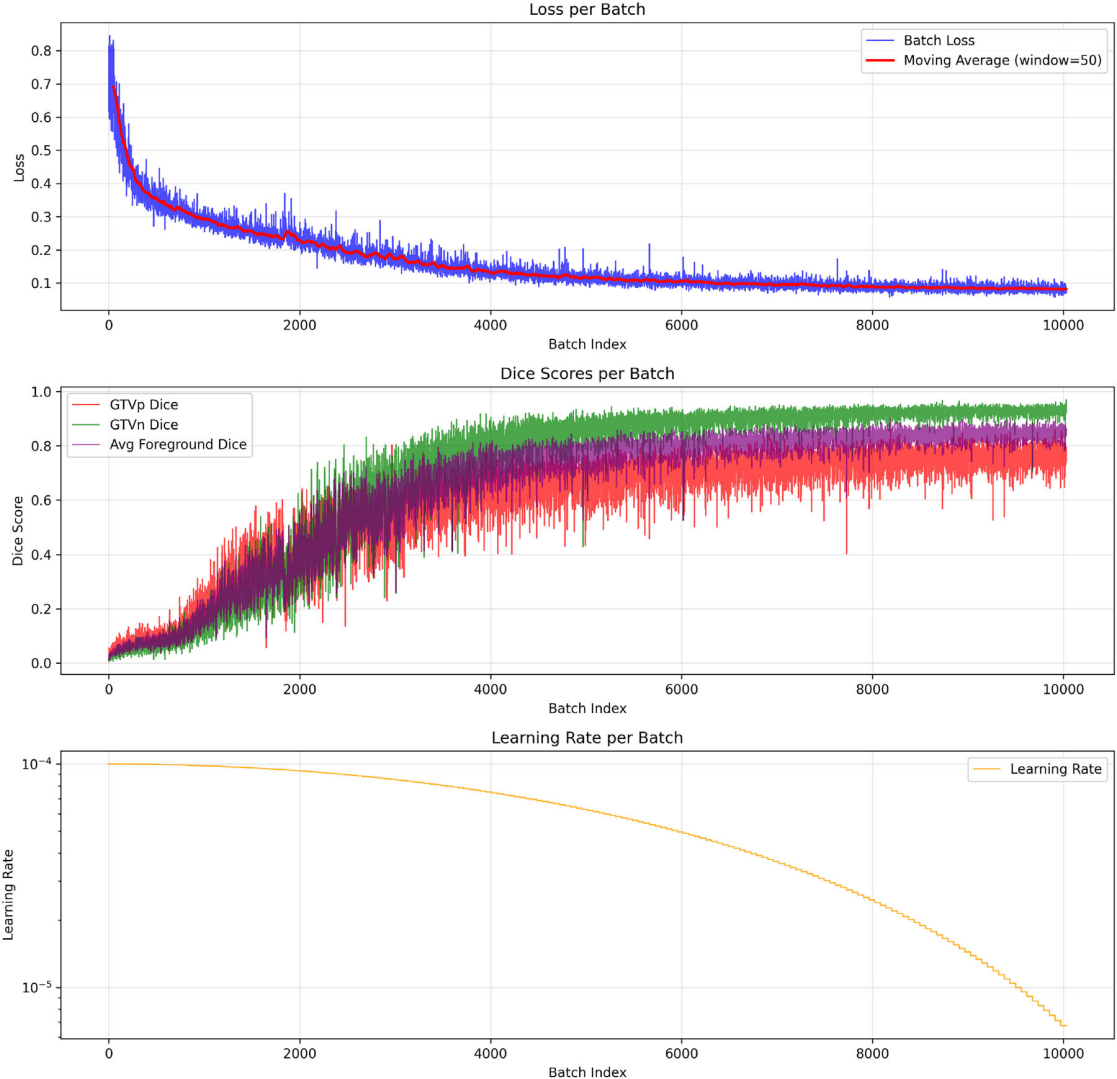
SCDU‐Net batch‐level training progress monitoring. Top panel: Training loss curve showing per‐batch loss values (blue) and their 50‐batch window moving average (red). Middle panel: Dice coefficient evaluation curves displaying the progression of GTVp (primary tumor volume, red), GTVn (nodal volume, green), and average foreground Dice scores (purple). Bottom panel: Learning rate decay curve (log scale) illustrating the learning rate scheduling strategy, starting from 1e‐4 and decreasing to 1e‐5 at 10 000 batches.GTVn metastatic lymph nodes; GTVp, primary gross tumor volume.

Figure 8 illustrates the evolution of loss values on both training and validation sets as a function of iteration number. Figure 9 demonstrates the variations of target GTVp and GTVn during the training process of the proposed model. As evident from the results, the loss function exhibits an overall decreasing trend throughout the training process, indicating that the network continuously optimizes its parameters to minimize prediction errors. For the SCDU‐Net model, the loss values stabilize at approximately the 200th iteration, suggesting that the model has essentially completed training and reached convergence at this stage, with no significant subsequent variations.

### 4.5 Experiment results

To evaluate the effectiveness of our proposed method, we implement several classical models on our self‐partitioned HNTS‐MRG datasets. Table 5 presents the quantitative comparison of our method against baseline approaches on the self‐partitioned test set. Statistical significance was evaluated using the Wilcoxon signed‐rank test with Bonferroni correction (α=0.0021). For the Dice coefficient, our method achieves 0.815 (GTVp), 0.853 (GTVn), and 0.834 (average), demonstrating significant improvements over nnU‐Net (p<0.001), SEU‐Net (p<0.001), and Deeplabv3 (p<0.001).

**TABLE 5:** Performance comparison on self‐partitioned test set from HNTS‐MRG dataset with statistical significance testing.

	DSC (↑)			Hausdorff95 (mm) (↓)		
Methods	GTVp	GTVn	Avg	GTVp	GTVn	Avg
nnU‐Net	0.5675 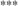	0.7214 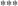	0.6444 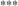	13.15 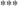	10.30 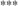	11.72 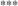
SEU‐Net	0.7163 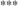	0.7945 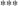	0.7554 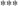	9.42 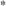	7.64 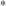	8.53 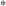
Deeplabv3	0.7236 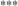	0.7911 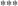	0.7573 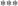	9.37 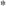	7.64 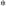	8.50 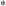
STU‐Net	0.8101	0.8394	0.8247	**6.96**	6.50	**6.73**
**Ours**	**0.8147**	**0.8532**	**0.8339**	7.35	**6.21**	6.78

Abbreviations: DSC, Dice similarity coefficient; GTVn metastatic lymph nodes; GTVp, primary gross tumor volume; HNTS‐MRG, head and neck tumor segmentation for MR‐guided applications.

*Note*: Bold values indicate the best performance for each metric. Statistical comparisons with our method using Wilcoxon signed‐rank test with Bonferroni correction: 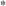 p<0.05, 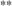 p<0.01, 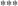 p<0.001 (significantly inferior to ours).

**FIGURE 10:**
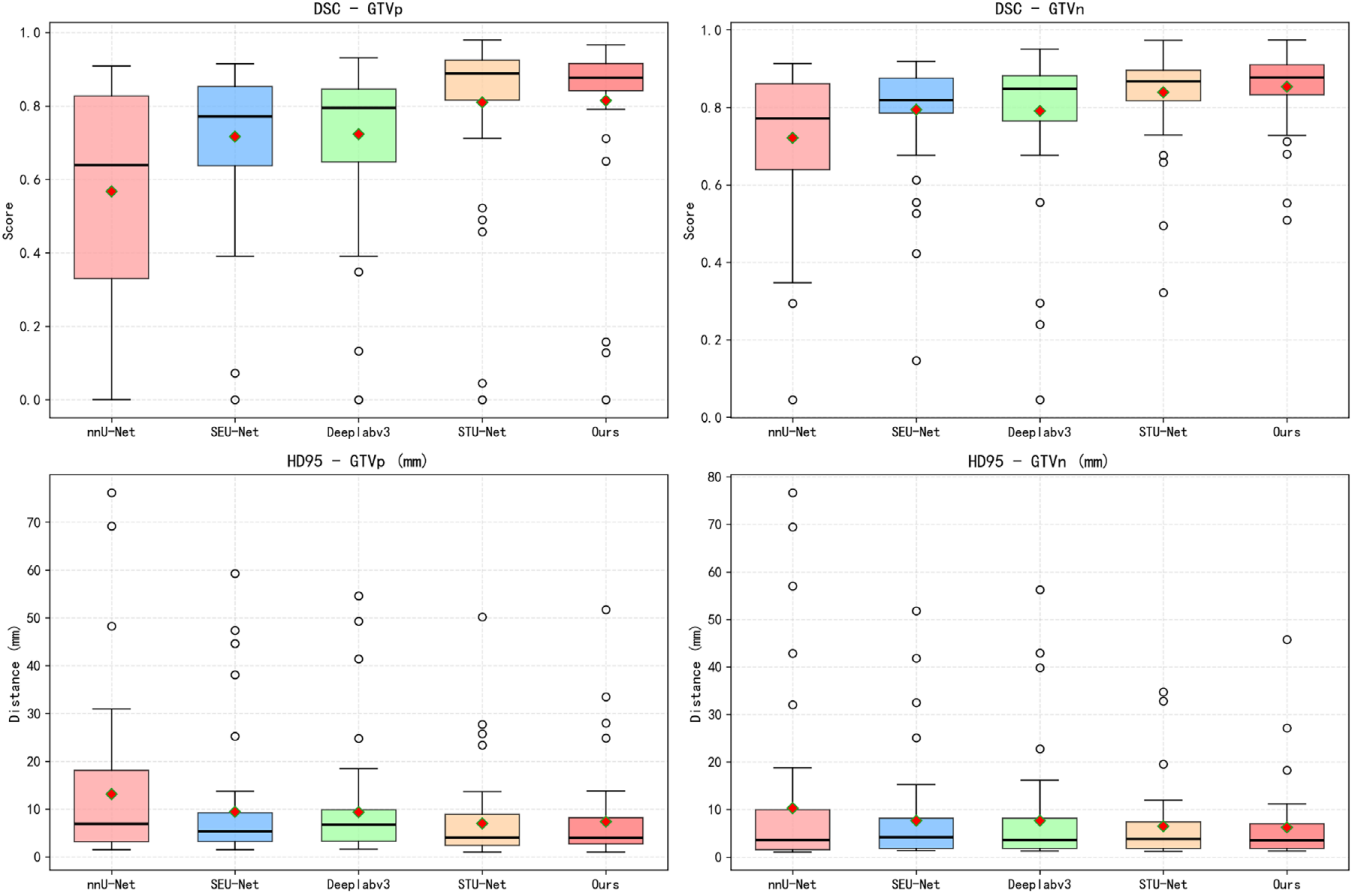
Boxplots comparing methods on Dice and Hausdorff95 metrics. Red diamonds: means; dots: outliers.

**FIGURE 11:**
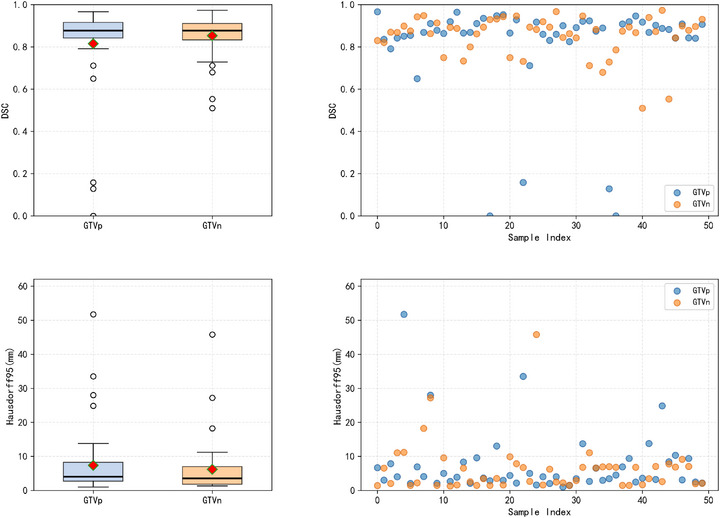
Boxplot and scatter plots of Dice coefficients and Hausdorff95 Distance. From the self‐partitioned test set.

For the Hausdorff95 metric, our method obtains competitive results with average distance of 6.78mm, comparable to STU‐Net's 6.73mm, while both methods outperform other baselines (p<0.01). These results indicate that our method effectively balances segmentation accuracy and boundary precision. We conducted a comparative analysis examining computational complexity and parameter efficiency for input images of size 448×448, with results presented in Table 6. Our model achieves a reasonable trade‐off between parameter count and segmentation effectiveness. The boxplots in Figure 10 compare various methods across Dice and Hausdorff distance metrics. Notably, despite the presence of outliers, our approach achieves performance comparable to STU‐Net in both median and mean values of DSC and HD95 metrics, suggesting stable learning performance when processing complex samples. Figure 11 displays the boxplots and scatter plots of both evaluation metrics on our self‐partitioned test set.

**TABLE 6:** Comparison of GFLOPs (Giga Floating‐point Operations Per Second) and number of parameters (M) among different models.

Methods	GFLOPs	Param. (M)
nnU‐Net	167.403	31.04
SEU‐Net	169.185	31.92
Deeplabv3	52.303	26.01
STU‐Net	510	58.26
Ours	181.89	45.82

Table 7 compares our model with other methods for head and neck segmentation. Iantsen et al.^[^ ^41^ ^]^ optimized U‐Net using patch‐wise normalization, scheduled data augmentation, and Gaussian weighted inference, achieving a DSC of 0.7490 on MRI. Wang et al.^[^ ^42^ ^]^ proposed a dual flow UNet with pre‐training and MixUp augmentation for multi‐timepoint MRI segmentation, achieving a DSC of 0.8238. Our method achieves a DSC of 0.8340, demonstrating competitive performance.

**TABLE 7:** Comparison results with other head and neck tumor segmentation methods. The optimal results for each metric are displayed in bold.

Methods	GTVp	GTVn	Average
lantsen et al.^[^ ^41^ ^]^	0.6950	0.8040	0.7490
L. Wang et al.^[^ ^42^ ^]^	0.7851	**0.8624**	0.8238
**Ours**	**0.8147**	0.8532	**0.8339**

Abbreviations: GTVn metastatic lymph nodes; GTVp, primary gross tumor volume.

*Note*: The optimal results for each metric are displayed in bold.

**FIGURE 12:**
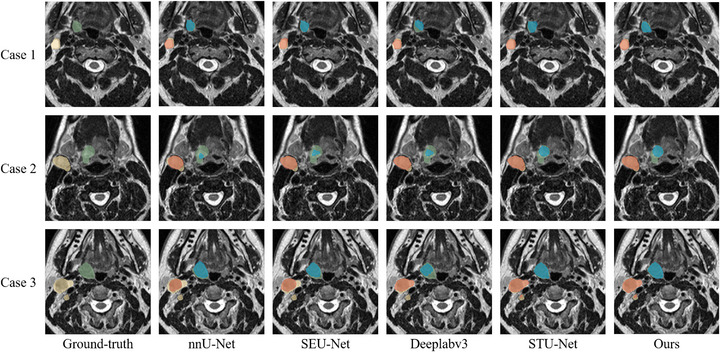
The segmentation results for several approaches are visualized. Ground‐truth: Green:GTVp, Yellow:GTVn; Predict: Blue: GTVp, Red: GTVn.GTVn metastatic lymph nodes; GTVp, primary gross tumor volume.

To visually demonstrate the segmentation performance of our model, tumor slices from representative patients are shown in Figure 12. The ground truth labels are color‐coded with green for GTVp and yellow for GTVn, while the predicted labels use blue for GTVp and red for GTVn.

## 5 CONCLUSION AND FUTURE WORK

This study proposes SCDU‐Net, which integrates SC and DenseASPP modules into the U‐Net framework for head and neck tumor segmentation. The model achieves acceptable overall segmentation performance with reduced computational demands through its 2D design. However, the 2D slice‐wise approach inherently lacks inter‐slice spatial context, which limits its effectiveness for small structures and volumetric consistency compared to 3D architectures. Additionally, the scarcity of publicly available head and neck MRI datasets restricts cross‐institutional validation. Clinically, SCDU‐Net can serve as an assistive tool in RT planning workflows, providing automated contouring suggestions for clinician‐identified regions of interest to improve delineation efficiency and consistency. Future work will focus on extending the architecture to 3D to better capture volumetric spatial dependencies while maintaining computational efficiency.

## AUTHOR CONTRIBUTIONS

**Qiang Han**: Conceptualization; methodology; software; formal analysis; investigation; data curation; writing‐original draft preparation; writing review and editing; visualization. **Songlin He**:Conceptualization; methodology; formal analysis; writing–review and editing; supervision. **Yuebin Zheng**:Conceptualization; methodlogy; formal analysis; writing review and editing, supervision. **Huacai Zhong**:Software; data curation. **Dengyao Luo**:Software. **Jun Wu**:Data curation. **Zhiqiang Zhao**:Software. **Bincheng Yan**:Data curation. **Chengjian Cao**:Conceptualization; supervision. **Xiu Liu**:Formal analysis; resources. Yuebing Deng and Qiang Han All authors have read and agreed to the published version of the manuscript.

## CONFLICT OF INTEREST STATEMENT

The authors declare no conflicts of interest.

## Data Availability

The original data presented in the study are openly available in the Multimodal Head and Neck tumor Segmentation Challenge 2024 repository at https://zenodo.org/records/11199559, accessed on 21 July 2025.
